# Study Protocol for a Yoga-Based Lifestyle Modification Program for Leucorrhea Disorders

**DOI:** 10.7759/cureus.78294

**Published:** 2025-01-31

**Authors:** Akanksha Mittal, Nibu R Krishna, Deepeshwar Singh, Vijaya Kavuri

**Affiliations:** 1 Department of Yogic Sciences, Lakshmibai National Institute of Physical Education, Gwalior, IND; 2 Department of Yoga, Babasaheb Bhimrao Ambedkar University, Lucknow, IND; 3 Department of Yoga, Vivekananda Yoga University (VaYU), Los Angeles, USA

**Keywords:** hormonal imbalance, leucorrhoea disorder, protocol, women’s health, yoga

## Abstract

Introduction: Leucorrhoea is natural vaginal discharge caused by hormonal changes, pregnancy, or sexual stimulation. This study protocol describes the methodology for a 12-week yoga program on the symptoms of leucorrhoea disorder among adult females.

Methods and analysis: The present study is a two-armed, randomized parallel-group, active-control trial for patients with blinded outcome assessors and multiple primary and secondary outcomes. The appropriate sample size will be determined based on the findings of the feasibility study. The participants will be recruited from a hospital and research centre in Madhya Pradesh, India. Individuals who meet the inclusion criteria for leucorrhoea disorder will be chosen following laboratory examinations. Randomization will be used to assign the chosen individuals to either the intervention or control groups (1:1). For adult females with leucorrhoea disorders, the intervention will consist of a yoga-based program that will run for one hour, five days a week, for 12 weeks. Data will be collected at baseline and post-intervention, in the 12th week. The outcome measures will include various primary (colour, odour, amount, vaginal culture, vaginal smear, and imbalance of various gonad hormones) and secondary (lower abdominal pain and quality of life) outcomes. The analysis will involve intention-to-treat and per-protocol approaches that will evaluate the impact of yoga on various outcome measures and will be assessed using statistical tests.

Ethics and dissemination: The study has been approved by the Institution Ethics Committee of Lakshmibai National Institute of Physical Education. Written informed consent will be obtained from each participant before inclusion. Results will be available through research articles and conferences. The summary of key results in layman's language will be made publicly available through newspaper articles.

## Introduction

Female reproductive capacity, mental health, the ability to work, and the ability to engage in regular physical activity are all significantly impacted by gynaecological problems. One of the main issues women face in the reproductive age range is leucorrhoea, a non-pathological vaginal discharge [1]. The majority of female patients seek gynaecological treatments because of excessive vaginal discharge; the causes range from physiological to pathological. Female patients will continue to experience distress until the underlying issue is identified and treated [2]. The female genital tract has a complex microbial flora, and the quantity and quality of cervical and vaginal secretions fluctuate based on factors such as age, menstrual cycle, and oral contraceptive use [3]. An abnormal vaginal discharge can be yellow, green, brown, or red, with a foul odour, pruritus, or dysuria depending on the cause of infection. There are many studies in developed nations that have indicated that up to 90% of vaginal discharge cases are caused by sexually transmitted infections (STIs) [4-5]. Due to excessive vaginal discharge, patients experience several issues, including anxiety, backache, and itching in their vulva. It is also linked to physical problems such as weakness, chronic fatigue, and soreness, and manifests as vague pain symptoms [6]. Some of the conditions connected to the symptoms are infertility, anaemia, lower genital tract infections (LGTIs), urinary tract infections (UTIs), acute pelvic inflammatory disease (PID), and problems with periods. These conditions can significantly impact women's reproductive health and overall well-being [7]. A community-based cross-sectional study of 506 females found 139 (27.47%) had leucorrhoea. A higher prevalence of leucorrhoea was observed in women who were married (35.29%), pregnant (28%), from a lower socioeconomic background, and had multiple pregnancies [8].

Leucorrhoea is a condition that has been researched in several Indian contexts, with an emphasis on women's views as well as the medical and clinical aspects. It is challenging for a researcher to determine the precise levels of prevalence because of the large folk lexicon and the perception of the disease's severity. While some believe it to be an incurable condition, the majority of women regard it as a typical occurrence in their lives. Often, cultural, societal, and other group-specific aspects impact the disease's normalcy. It might be pathological or physiological in adult women. The pathology can be classified as infectious (owing to one or more infections) or non-infectious (detergents, foreign bodies, herbal remedies, or due to some tumours). Premenstrual syndrome, pregnancy, or sexual arousal are possible causes of the physiological changes. According to its biological characteristics, leucorrhoea is linked to both STIs and localized illnesses of the reproductive system. This condition is known by several regional names throughout India due to cultural differences and is taken seriously even in cases where the discharge is not pathological [9]. Vaginal discharge is significantly influenced by hormonal regulation, with estrogen and progesterone playing key roles in its production and consistency. Female genital secretion research remains significantly understudied, representing a critical gap in comprehensive reproductive biological investigation. However, recent studies have begun to shed light on the importance and potential benefits of further understanding women's genital secretions [10-11].

Yoga is an ancient practice that focuses on the mind and body, and it has been shown to improve both the physical and mental health of women. Researchers have found that yoga can improve blood circulation and hormonal balance, which can potentially enhance the quality and quantity of women's genital secretions. Additionally, certain yoga poses and exercises specifically target the pelvic floor muscles, which can improve overall vaginal health and possibly increase natural lubrication [12]. Regular yoga practice has been shown to alleviate menstrual cramps and reduce the duration and intensity of menstrual cycles. Moreover, it regulates hormonal imbalances that help to reduce the excessive white vaginal discharge [13]. By strengthening the pelvic floor muscles, yoga can also aid in preventing and treating urinary incontinence, a common issue for many women. However, distinct perception-based and cultural barriers have been identified that influence women’s participation in yoga to improve their health [14]. Regular yoga practice has been shown to improve physical and psychological well-being and balance the neuro-endocrine system, which is beneficial for the menstrual cycle and pregnancy [15]. Yoga practices can diminish perceived stress and anxiety and improve sleep quality in patients suffering from dysfunctional uterine bleeding, thereby improving their quality of life [16].

Conventional treatments often neglect underlying psychosomatic and lifestyle factors. Yoga, known for its holistic benefits in promoting hormonal balance, reducing stress, and improving pelvic health, offers a promising non-invasive approach. This study hypothesizes that a 12-week yoga-based lifestyle intervention can effectively reduce leucorrhoea symptoms, enhancing overall reproductive and sexual well-being in women.

The purpose of this study is to evaluate the efficacy of yoga practices as a therapeutic technique for the treatment of leucorrhoea disorders in adult females.

## Materials and methods

Study design

**Figure 1:**
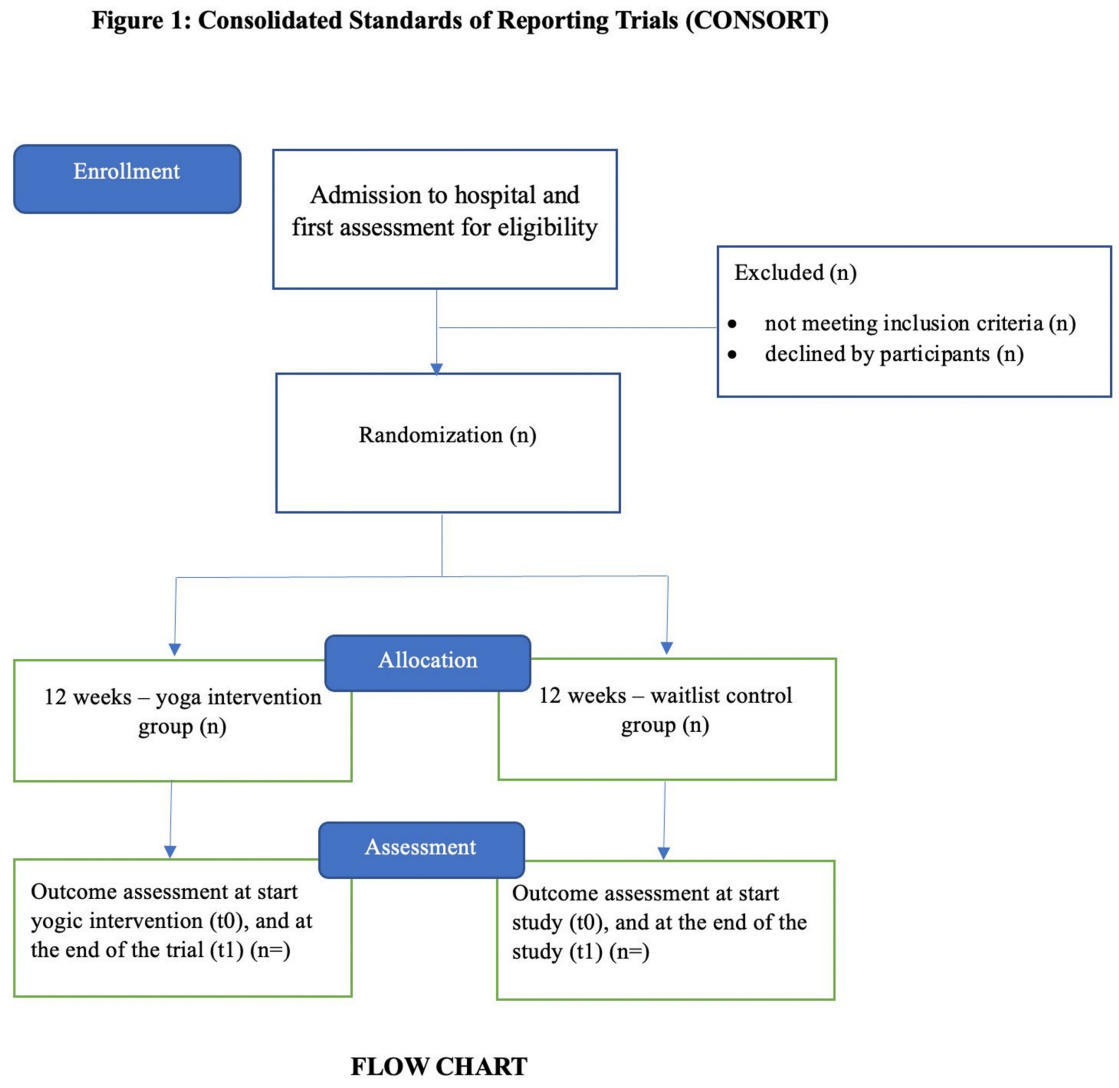
CONSORT flow diagram.

The present study will be a two-armed, randomized parallel-group, active control trial for patients with blinded outcome assessors and multiple primary and secondary outcomes. The method of generating random sequences is computer-generated randomization, and the method of concealment is sequentially numbered, sealed, opaque envelopes. Yoga is a lifestyle intervention that has a holistic impact on diverse domains of health, including physiological, metabolic, psychological, and social wellness. The goal of the study is to facilitate a healthy reproductive life for women by using yoga as a lifestyle intervention. The Consolidated Standard of Reporting Trials (CONSORT) model was utilized in the process of drafting the protocol. The participants will be recruited from a gynaecological hospital (Dr. Kaul Hospital and Research Centre) in Gwalior, Madya Pradesh, India. December 2024 marked the beginning of the recruitment process, which included the distribution of leaflets and visits to hospitals and gynaecological facilities. Our goal is to finish collecting the data by the month of December 2024. This study will conduct a screening to identify participants who are suffering from leucorrhoea and then ask them to participate in the research. Those who do not meet the eligibility requirements will be excluded from participation. Figure 1 contains the criteria for inclusion and exclusion to be considered. Informed written consent would be obtained from participants who volunteered their participation.

Randomization and blinding

An impartial statistician who is not involved in the research will carry out the randomization and blinding procedures for the study. During the baseline visit, a sequence randomizer will be used to randomly assign participants to one of two groups, with each group consisting of 40 individuals using a ratio of one to one. Following the completion of the baseline evaluations, the participants will be notified about the future procedure, and the allocation sequences will be sealed once they have been completed. Due to the nature of the intervention, it is not possible to conduct a comprehensive procedure for double-blinding. All investigators who will be involved in the evaluation of the outcome measures will be blinded to the randomization groups. This could help to limit the likelihood of bias being detected. 

Search strategy

A systematic search will be performed in six English databases (i.e., PsycINFO, Embase, Cochrane Library, CINAHL, Web of Science, and PubMed), which will help ensure a comprehensive search that will include as much relevant literature as possible. The inclusion criteria will consist of the following conditions: Patients will be selected based on a wet vaginal smear and observation by a doctor. The participants will include infertile women. Additionally, the presence of Trichomonas vaginalis, fungal hyphae, or pus cells in the wet vaginal smear will be required for inclusion. Participants' age criteria should be restricted to 18 to 35 years. Diabetic patients and PCOD patients will be included. Participants who have undergone surgeries such as C-section delivery and tubal ligation will also be included. Moreover, patients suffering from STDs like syphilis and gonorrhoea, as well as those with congenital abnormalities or other pathologies of the reproductive tract, will be considered for the study. The exclusion criteria will be as follows: pregnant women, patients suffering from neurocognitive disorders, physically handicapped patients, and those who regularly consume steroid drugs, contraceptive pills, or Viagra pills.

Data collection

Randomization will be performed between two groups of participants. The vaginal discharge assessment form should be filled out by all the participants. After obtaining written consent from the participants and confirmation through a lab report by the gynaecologist about the patient's condition, pre-testing will be conducted, and data will be collected on the selected variables. The obtained data will be tabulated as baseline data. Before starting the intervention program, an interactive introductory lecture will be conducted. In the introductory lecture, the purpose and design of the study will be explained to the participants. A structured yoga module for managing leucorrhoea was developed, incorporating yoga postures, breathing techniques, meditation, and relaxation practices. The module was validated by 20 experts, who recommended the most effective practices based on their expertise. This validated protocol aims to provide a holistic, non-invasive approach to managing leucorrhoea symptoms (such as lower abdominal pain, lower back pain, fatigue, weakness, etc.). Yoga practices will be taught to the participants for 12 weeks. After the completion of three months of intervention, the lab reports and psychological assessments will be collected for the second time by the researcher, and the data will be tabulated for the statistical procedure.

Yoga intervention procedure

The yoga intervention will involve weekly group sessions, conducted five days a week for 60 minutes, from 6:00 AM to 7:00 AM. Participants will learn and practice specific yoga poses, breathing exercises, and meditation techniques tailored to address the symptoms and concerns associated with leucorrhoea. The sessions will be led by a certified yoga instructor, with instructional materials and resources provided to support home practice. This intervention aims to promote physical and mental well-being, reduce stress levels, and improve the overall quality of life for individuals with leucorrhoea. The waitlist control group will not receive any intervention during the study period but will be offered the yoga program after the study’s completion, based on their interest. The description of yoga practices is given in Table 1 [17-19].

**Table 1:** Description of Yoga Practices.

Yogic Practices	Timing/Rest	Description
Manglacharan (opening prayer): "Oṁ Saha nāvavatu, saha nau bhunaktu, Saha vīryam karavāvahai Tejasvi nāvadhītamastu Mā vidviṣāvahai, Oṁ Shāntiḥ, Shāntiḥ, Shāntiḥ [17]."		Prayer should be chanted followed by explaining the meaning. Translation: Om, May we all be protected, May we all be nourished, May we work together with Great energy, May our intellect be sharpened, (and may our study be effective) Let there be no animosity amongst us. Om, peace (in me), peace (in nature), peace (in divine forces).
Pawanmuktasana Part 1: Anti-Rheumatic Group (loosening practices)		This group of asanas is concerned with loosening up the joints of the body. It is excellent for those with rheumatism, arthritis high blood pressure, heart problems or other ailments where vigorous physical exercise is not advised. It is particularly useful for eliminating energy blockages in the joints and outer extremities of the physical body and works on the physical and mental bodies as well. It is a gentle and effective practice for those individuals who may need a more gentle approach to physical activity [18].
- Goolf ghoornan (ankle crank)	3 rounds × 90 sec; 10 sec rest	All foot and calf asanas help in returning the stagnant lymph and venous blood. They thus relieve tiredness and cramps and prevent venous thrombosis, especially in bedridden, post-operative patients [18].
- Janu chakra (knee crank)	3 rounds × 90 sec; 10 sec rest	This area is the most vulnerable to injuries, sprains and osteoarthritis. All knee asanas strengthen the quadriceps muscle and the ligaments around the knee joint. These asanas rejuvenate the joint by activating the healing energies [18].
- Ardha titali asana (half-butterfly pose)	3 rounds × 90 sec; 10 sec rest	This is an excellent preparatory practice for loosening up the knee and hip joints for meditative poses. Those people who cannot sit comfortably in cross-legged positions should practise this pose daily, both morning and evening [18].
Pawanmuktasana Part 2: Digestive/Abdominal Group		This group of asanas is concerned specifically with strengthening the digestive system. It is excellent for those persons suffering from indigestion, constipation, acidity, excess wind or gas, lack of appetite, diabetes, disorders of the male or female reproductive systems and varicose veins. It also eliminates energy blockages in the abdominal area [18].
- Supta pawanmuktasana (leg lock pose)	3 rounds × 90 sec; 10 sec rest	This asana improves digestion, relieves bloating, acidity, and constipation, and enhances metabolic and reproductive health. It strengthens the lower back, loosens spinal vertebrae, and massages abdominal and pelvic organs, benefiting circulation, fertility issues, menstrual health, and eliminating energy blockages in the abdomen [18].
- Shava udarakarshanasana (universal spinal twist)	3 rounds × 90 sec; 10 sec rest	Tightness and tiredness are relieved, especially in the lower back. The pelvic and abdominal organs are toned through its massaging action [18].
Pawanmuktasana Part 3; Shakti bandha asanas (energy bock postures)		This group of asanas is concerned with improving the energy flow within the body and breaking down neuro-muscular knots, especially in the pelvic region where energy tends to stagnate. It is especially useful for menstrual problems and toning the pelvic organs and muscles. It can be practised before and after pregnancy, facilitating the process of childbirth and re-tuning flaccid muscles. These asanas also eliminate energy blockages in the spine, activate the lungs and heart, and improve endocrine function [18].
- Chakki chalanasana (churning the mill)	3 rounds × 90 sec; 10 sec rest	This asana is excellent for toning the nerves and organs of the pelvis and abdomen. It is very useful for regulating the menstrual cycle [18].
- Nauka sanchalan asana (rowing the boat).	3 rounds × 90 sec; 10 sec rest	This asana has a positive effect on the pelvis and eliminates energy blockages in this area. It is especially useful for gynaecological disorders [18].
- Kashtha takshanasana (chopping wood).	3 rounds × 90 sec; 10 sec rest	This asana loosens up the pelvic girdle and tones the pelvic muscles. It is useful for women preparing for childbearing and may be practised during the first three months of pregnancy [18].
Surya namaskara	5 rounds of 3 min each (3 dynamic and 2 slow), 1 min rest	Surya namaskar is a complete sadhana, spiritual practice, in itself for it includes asana, pranayama, mantra and meditation techniques. Regular practice of Surya namaskara regulates pingala nadi, whether it is under-active or over-active. Regulation of pingala nadi leads to a balanced energy system at both mental and physical levels [18].
Vajrasana (thunderbolt pose)	3 rounds × 60 sec; 10 sec rest	The Sanskrit word "vajra" refers to the nerve and energy pathway that connects the sexual organs to the brain. Vajrasana alters the flow of blood and nervous impulses in the pelvic region and strengthens the pelvic muscles. It reduces the blood flow to the genitals, assists women in labour and helps alleviate menstrual disorders. It also improves digestion and aids in relieving constipation [18]. It is helpful in preventing the loss of vitality from genital organs in leucorrhoea disorder [19].
Siddhyoni asana (accomplished pose for women)	3 rounds × 60 sec; 10 sec rest	This asana applies pressure to the pubic bone, pressing the trigger point for Swadhisthana, automatically activating Vajroli/Sahajoli mudra. These two psycho-muscular locks redirect sexual nervous impulses back up the spinal cord to the brain, establishing control over the reproductive hormones which is necessary to maintain brahmacharya for spiritual purposes. Prolonged periods in this asana result in noticeable tingling sensations in the Mooladhara region which may caused by a reduction in the blood supply to the area and by a rebalancing of thepranic flow in the lower chakras [18].
Paschimottanasana (back stretching pose)	3 rounds × 60 sec; 10 sec rest	It tones and massages the entire abdominal, pelvic region and adrenal glands. It helps alleviate disorders of the urogenital system [18].
Ushtrasana (camel pose)	3 rounds × 60 sec; 10 sec rest	This asana is beneficial for the digestive and reproductive systems. It also regulates the thyroid gland [18].
Shashankasana (pose of the moon/ hare pose)	3 rounds × 60 sec; 10 sec rest	It tones the pelvic muscles and the sciatic nerves and is beneficial for women who have an underdeveloped pelvis. It also regulates the functioning of the adrenal glands. It helps to alleviate disorders of both the male and female reproductive organs. It can be a helpful exercise for overall pelvic health [18]. It is helpful to balance the hormones in patients experiencing leucorrhoea [19].
Bhujangasana (cobra pose)	3 rounds × 60 sec; 10 sec rest	This asana gently tones the female reproductive organs, alleviates menstrual disorders and is an excellent asana for strengthening and tightening the pelvic region [18].
Shalabhasana (locust pose)	3 rounds × 60 sec; 10 sec rest	The parasympathetic nerves are particularly prominent in the regions of the neck and pelvis. Shalabhasana stimulates the whole autonomic nervous system, It strengthens the lower back and pelvic organs [18]. It is helpful to prevent the loss of vitality from genital organs in leucorrhoea disorder [19].
Dhanurasana (bow pose)	3 rounds × 60 sec; 10 sec rest	The pancreas and adrenal glands are toned, balancing their secretions. This leads to improved functioning of the digestive, eliminative and reproductive organs [18].
Marjariasana (cat stretch pose)	3 rounds × 60 sec; 10 sec rest	This pose gently tones the female reproductive system. Women suffering from menstrual disorders and leucorrhea will obtain relief by doing marjari-asana [18].
Matseyasana (fish pose)	3 rounds × 90 sec; 10 sec rest	The pelvic region is given a good stretch and the pressure of the feet on the thighs greatly reduces blood circulation in the legs, diverting it to the pelvic organs. This helps prevent and remove disorders of the reproductive system [18].
Ardhachakrasana (half wheel pose)	3 rounds × 90 sec; 10 sec rest	This pose is beneficial to the nervous, digestive, respiratory, cardiovascular and glandular systems. It influences all the hormonal secretions and relieves various gynaecological disorders [18].
Sarvangasana (shoulder stand pose)	3 rounds × 90 sec; 10 sec rest	This pose is used in yoga therapy for the treatment of menopause, menstrual disorders and leucorrhoea [18].
Vipareetakarani mudra (inverted psychic attitude)	3 rounds × 90 sec; 10 sec rest	The inverted posture sustained in this mudra is used to reverse the downward and outward movement of energy and redirect it back to the brain [18].
Pranayamas (breath techniques)		
Ujjayi pranayama (psychic breath)	3 rounds × 90 sec; 10 sec rest	This practice alleviates fluid retention. It removes disorders of the data, which are the 7 constituents of the body: blood, bone, marrow, fat, semen, skin and flesh [18].
Naadishodhan (psychic network purification)	3 rounds × 90 sec; 10 sec rest	This practice ensures that the whole body is nourished by an extra supply of oxygen. It increases vitality and lowers levels of stress and anxiety by harmonising the pranas [18].
Bandhas (binds/contractions)		
Moola bandha (perineum contraction)	3 rounds × 90 sec; 10 sec rest	It stimulates the pelvic nerves and tones the urogenital and excretory systems. Moola bandha is both a means to attain sexual control (brahmacharya) and to alleviate a multitude of sexual disorders. It enables sexual energy to be directed either upward for spiritual development [18].
Uddiyana bandha (abdominal contraction)	3 rounds × 90 sec; 10 sec rest	It creates a suction pressure that reverses the flow of the sub-pranas, apana and prana, uniting them with Samana. there is an explosion of subtle force that travels upward through Sushumna Nadi [18].
Concentration on Swadhisthana chakra	5 min	Swadhisthana is associated with the organs of excretion and reproduction. Visualisation of this centre can help rectify disorders of these functions [18].
Closing prayer: "Asato mā sad gamaya, tamaso mā jyotir gamaya, mṛtyor māmṛtaṁ gamaya" [17].		Prayer should be chanted followed by explaining the meaning. Translation: "Lead us from ignorance to knowledge, from darkness to light and from death to immortality."

Measures to maintain adherence

Yoga classes will be provided free of charge and conducted within a radius of 1 km from the hospital. The yoga practices will be implemented throughout the study duration, with participants receiving regular reminders and support from the researcher to encourage adherence to the prescribed regimen. Weekly check-ins will be conducted to address any concerns, resolve difficulties, and offer guidance as needed. Attendance will be recorded for all sessions to monitor compliance. The intervention consists of 12 weeks of yoga sessions, conducted five days a week, totalling 60 sessions. A minimum attendance of 48 sessions (80% adherence) will be required to include participants’ data in the analysis. These measures aim to ensure a high level of adherence and maximize the intervention’s effectiveness in managing leucorrhoea.

Outcome measures

The research personnel will conduct screening for potential participants. Further participants will be asked to report any adverse events, i.e., injury resulting from the program, and will be advised to consult their physician to provide care as appropriate.

Participants will undergo various quantitative assessments, including biochemical tests, urine analysis, vaginal examinations, and blood tests such as glycated haemoglobin (HbA1c) and VDRL tests. Routine urine testing and microscopic examinations will be conducted both pre- and post-treatment. Additionally, the quality of life will be evaluated using the SF-36 (Short Form Survey) Questionnaire. Lower abdomen discomfort will be measured using a VAS (Visual Abdominal Pain Analog Scale). Every patient will have vaginal pH (Potential of hydrogen) test and a vaginal wet smear performed both before and after the yoga intervention. If necessary, a vaginal swab culture test will be performed.

Vaginal pH Test

A vaginal pH test can tell how acidic or alkaline (basic) the vaginal secretions are. An acidic vagina is indicated by a pH range of 3.8 to 4.5, which is moderately acidic. A lower pH value (more acidic) in the vagina than the blood or interstitial fluids can protect the vaginal mucosa from pathogenic organisms. If a woman experiences unusual vaginal symptoms such as burning, irritation, foul odour, or abnormal vaginal discharge, she may need to get her vaginal pH checked. Vaginal kits include vaginal pH strips, which will be inserted in the vagina. The vaginal pH determines the colour of the paper; various pH ranges result in distinct colours. Any infections that can be connected to vaginal pH should be diagnosed by a physician.

Vaginal Wet Smear

The purpose of a vaginal wet mount, sometimes called a vaginal smear, is to identify the source of vaginitis, or inflammation of the vulva and vagina. Vaginal irritation is frequently caused by infection. The most common infections that result in vaginitis are trichomoniasis, yeast infections, and bacterial vaginosis. The wet mount technique involves placing a sample of vaginal discharge on a slide. White blood cells, yeast, bacteria, and trichomoniasis are then detected by testing and microscopic examination of the sample [20]. To locate the source of vaginal burning, irritation, redness, odour, or discharge, utilize a vaginal wet mount.

VDRL

This test is intended to diagnose syphilis and STIs in individuals. Syphilis is brought on by the bacterium *Treponema pallidum*. The bacterium penetrates the mouth or vaginal region and multiplies [21]. The VDRL test searches for antibodies that the body makes in response to antigens present in cells that have been harmed by bacteria. By looking for these antibodies, doctors may be able to diagnose you with syphilis. For the VDRL test, a medical professional will take a blood sample from you. Usually, a vein at the back of the hand or in the crease of the elbow is used to draw blood. Next, this blood sample will be examined in a lab to check for syphilis-related antibodies.

HbA1c

The haemoglobin A1c test measures the amount of blood sugar (glucose) attached to haemoglobin. It is well-established that diabetes mellitus is associated with an increased risk of infections. An epidemiologic study suggests that women with diabetes, especially those with poorly controlled blood glucose (hyperglycemia), have a greater risk of LGTIs including vulvovaginal candidiasis [22]. Several studies show an association between diabetes and UTI in women [23-24]. A sample of blood is taken from an arm vein and placed in a test tube or vial to conduct the HbA1c test.

Oestrogen Test

The level of estradiol, a kind of oestrogen hormone, in the blood is determined by an E2 (estradiol) test or estrone blood test. The primary and most potent type of oestrogen in the body is called estradiol, or E2. It is essential for growing and maintaining female reproductive tissues as well as for controlling the menstrual cycle, ovulation, follicle development, pregnancy, bone health, and general well-being. A pathologist will take a blood sample from a vein in the arm using a small needle. After the needle is inserted, a small amount of blood will be collected into a test tube or vial. This usually takes less than five minutes.

Progesterone Test

A progesterone test is a blood test used to measure progesterone levels, which are crucial for many aspects of reproductive health. Normal vaginal discharge changes in response to hormonal changes from both ovarian hormones, estrogen and progesterone. The level of these circulating hormones changes throughout the menstrual cycle and changes the quality and quantity of discharge [25]. The progesterone test, typically conducted around day 21 of the menstrual cycle, is used to assess luteal phase hormone levels, which can help identify any hormonal imbalances. This information is critical for understanding the hormonal profile of participants and ensuring that the observed symptoms are related to leucorrhoea rather than other underlying hormonal disorders. 

Urine Examination

A urine sample is collected to test for a vaginal infection. The sample is examined by a pathologist who will determine if bacterial overgrowth has occurred and resulted in a vaginal bacterial infection. The test kits include a disposable vaginal swab, a urine sample kit, or both. The sample will be examined to determine the pathogens (bad microorganisms) that may be causing your symptoms and offer suitable recommendations regarding treatment regimes.

Quality of Life

Recurrent leucorrhoea can significantly impair a woman’s quality of life by causing physical discomfort, emotional distress, and social embarrassment. The SF-36 questionnaire is a well-researched, self-reported tool to measure overall health conditions. It is based on the medical outcomes study, which sought to objectively assess quality of life. The SF-36 was created by its original authors to assess individual health in clinical practice and research, as well as population health in health policy reviews and general population surveys. It has been utilised in thousands of research investigations. Leucorrhoea is often associated with symptoms such as excessive vaginal discharge, itching, lower abdominal pain, and fatigue. These symptoms may lead to anxiety, sleep disturbances, and difficulty in performing daily activities. Additionally, the persistent nature of the condition can contribute to a reduced sense of well-being and negatively impact mental health [26]. The SF-36 was created as a general health measure, but it has also been used to assess specific disease populations [27].

Lower Abdominal Pain

Some women with leucorrhea disorder also have complaints of lower abdominal pain. Additionally, other female reproductive disorders can cause pelvic pain, and those conditions need to be ruled out. The VAS is a tool used to measure pain intensity by asking patients to mark a point on a line that represents their level of pain. The VAS will measure the intensity of the lower abdominal pain. It is one of the pain rating scales used for the first time in 1921 by Hayes and Patterson. It is commonly used in epidemiologic and clinical studies to assess the severity or frequency of certain symptoms. For example, a patient's pain level can range from none to intense. From the patient's perspective, this spectrum appears continuous; their pain does not occur in discrete jumps, as a categorization of none, mild, moderate, and severe would imply. The VAS was designed to capture the concept of an underlying continuous. The pain VAS is a one-dimensional measure of pain intensity that is used to track patients' pain progression or to compare pain severity among individuals with similar medical conditions [28]. 

Statistical techniques

Sample Size Estimation

Conducting the first study on the effects of yoga on patients with leucorrhoea is a significant step toward understanding potential therapeutic interventions for this condition. This would be a prudent approach to ensure that our research is both scientifically rigorous and practically achievable. The sample size will be calculated using G Power Software for paired comparison, including a power of 80%, an alpha value of 0.05, a two-sided test and an attrition rate of 20% [29]. The laboratory investigations will be done on eligible participants who satisfied the inclusion criteria for leucorrhoea disorder. The collection of data is completely dependent upon the performance of all these specific yogic interventions by the experimental group for 12 weeks. The sample will be tested twice (pre- and post-test) with an interval of a minimum of 12 weeks of training. There will be two groups: (a) the experimental group and (b) the waitlist control group.

Data Analysis

The evaluation will primarily involve descriptive analysis to summarize the baseline characteristics and outcomes. To assess the effectiveness of the intervention, a global statistical test (GST) will be applied. The pre-treatment characteristics of the sample will be compared with those of the target population, and a per-protocol analysis will be conducted.

For continuous variables (e.g., age, hormone levels, and duration of symptoms), the mean and standard deviation (SD) or median and interquartile range (IQR) will be used to summarize the baseline data. A comprehensive examination of the treatment’s objectives will also be performed. Missing data will be addressed using various imputation techniques, and sensitivity analyses will be conducted to assess the robustness of the results. To estimate log-binomial models, generalized estimating equations (GEE) will be applied, while mixed-effects linear regression models will be used to analyze the data.

Outcomes will be evaluated both at baseline and after 12 weeks, with all available data considered in the analysis. Statistical analyses will be performed using IBM SPSS, Version 21, and JASP. A significance level of p<0.05 will be applied throughout the analysis.

Ethical statement

The study protocol is approved by the Institutional Ethics Committee of Lakshmibai National Institute of Physical Education, Gwalior issued approval IEC/LNIPE/01/2022/05. The trial has been registered in the Clinical Trial Registry of India - CTRI/2022/11/047474, and the study was conducted in accordance with the principles of the Declaration of Helsinki. 

The consent of participants in the research study will be obtained, and their privacy and confidentiality will be maintained throughout the study. Any potential risks or discomforts associated with the study will be minimized, and participants will be provided with adequate information to make an informed decision about their participation. The results of the study will contribute to a better understanding of the effectiveness of yoga in managing leucorrhoea disorders and may have implications for future treatment options.

Confidentiality

The confidentiality of the participants will be a top priority, and all data collected will be kept strictly confidential. Only the researchers involved in the study will have access to the information, and any identifying details will be removed to ensure anonymity. Additionally, all data will be stored securely and destroyed after the completion of the study. This commitment to privacy and confidentiality will help to establish trust with the participants and encourage open and honest responses, ultimately leading to more reliable and valid results.

## Results

After completing data collection and analysis, the results will be systematically compiled and presented in a clear and concise manner. Descriptive analysis will be used to summarize the baseline characteristics of the participants, with continuous variables (e.g., age, hormone levels, and duration of symptoms) being summarized using the mean and standard deviation (SD) for normally distributed data or median and interquartile range (IQR) for non-normally distributed data. Categorical variables will be presented using frequency counts and percentages. The effectiveness of the intervention will be assessed using a global statistical test (GST) to compare the pre-treatment features of the sample with those of the target population. A per-protocol analysis will be conducted to evaluate the intervention’s impact on outcomes, including changes in symptoms and quality of life.

Missing data will be addressed using appropriate imputation techniques, and sensitivity analyses will be conducted to ensure the robustness of the results. To estimate log-binomial models, generalized estimating equations (GEE) will be used. Additionally, mixed-effects linear regression models will be applied to assess the impact of the intervention, with outcomes measured at both the baseline and after 12 weeks. Results will be shared with participants and stakeholders, and the findings will be disseminated through academic publications and conference presentations to maximize the study’s impact and contribution to the field.

## Discussion

The present study aims to investigate the effectiveness of a yoga-based lifestyle modification program on managing leucorrhoea. By incorporating both biochemical and qualitative assessments, the trial will provide a comprehensive evaluation of yoga’s impact on key indicators such as hormonal balance, inflammation, and the frequency and severity of symptoms. Participants will be carefully selected based on confirmed diagnoses of leucorrhoea, ensuring homogeneity of the sample. The experimental group will practice a structured yoga program, including asanas and breathing exercises, while the control group will maintain their usual lifestyle, serving as a comparative baseline.

**Figure 2:**
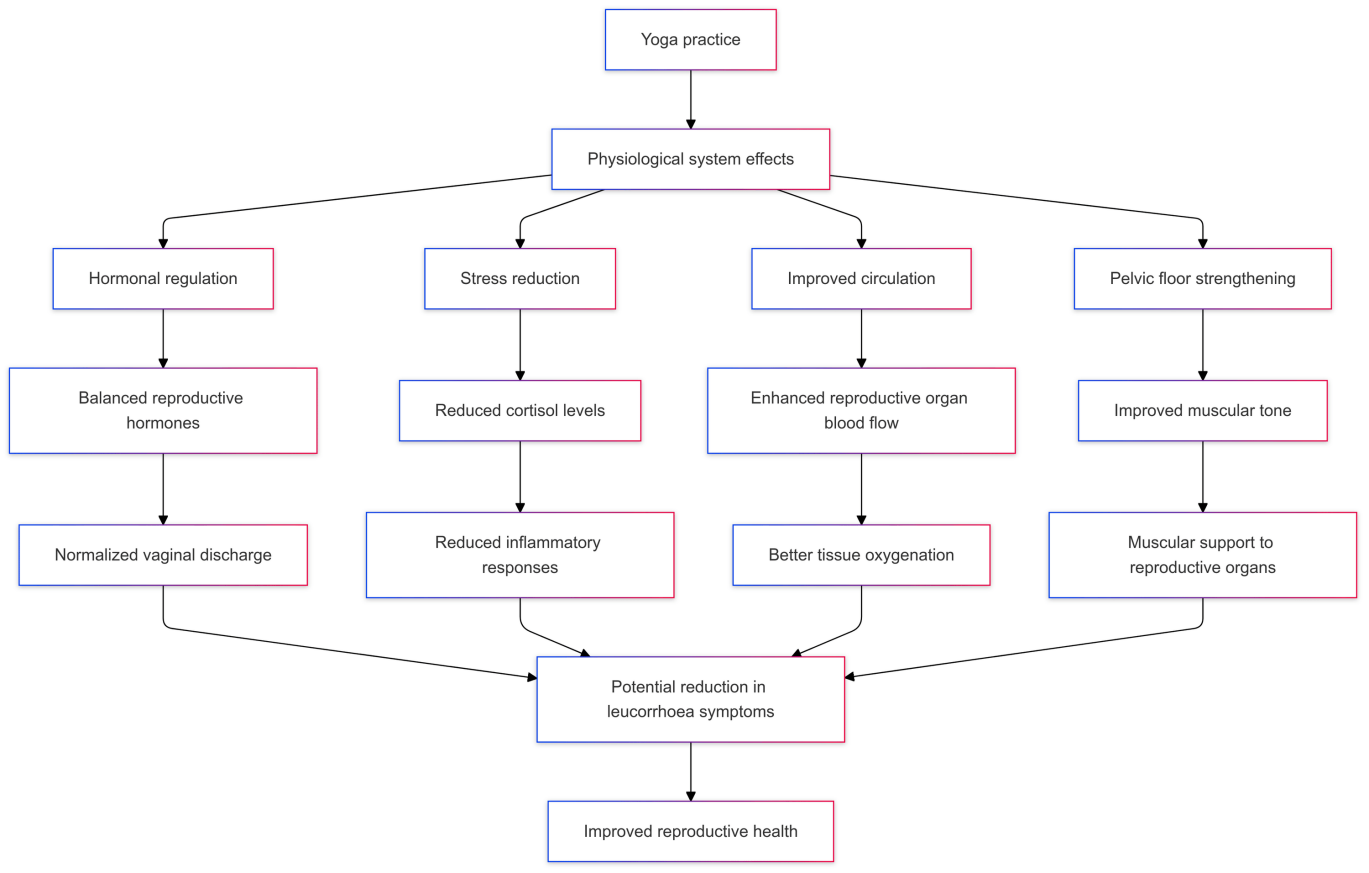
Hypothetical mechanism of action for the effectiveness of yoga in managing leucorrhoea: stress reduction, circulatory enhancement, hormonal balance, and pelvic floor strengthening

The hypothetical mechanism of action for yoga’s effectiveness includes stress reduction, improved circulation, hormonal balance, and pelvic floor muscle strengthening, all of which are interconnected. These mechanisms are hypothesized to improve vaginal health, reduce abnormal discharge, and alleviate associated symptoms. This integrative approach, illustrated in the flowchart, aligns with the holistic nature of yoga and emphasizes its potential as a non-invasive treatment option.

The COVID-19 pandemic has heightened health awareness, encouraging women to seek proactive approaches to well-being [30]. During recruitment, participants meeting the inclusion criteria will be informed about the holistic benefits of yoga and encouraged to participate consistently throughout the study. This emphasis on informed consent and patient engagement is expected to enhance adherence and outcomes. By combining robust methodology with evidence-based yoga practices, this trial has the potential to contribute significantly to the field of complementary therapies for women’s health. The study design ensures validity and reliability, making it a valuable framework for future research. The results are anticipated to advance understanding of yoga’s role in managing leucorrhoea and promote its practical applications in reproductive health care.

## Conclusions

This protocol establishes a framework to assess the efficacy of yoga practices in managing leucorrhoea, aiming to improve symptoms and well-being while contributing valuable insights into women’s health and complementary therapeutic approaches.
